# Chagas Cardiomyopathy in the Context of the Chronic Disease Transition

**DOI:** 10.1371/journal.pntd.0000688

**Published:** 2010-05-18

**Authors:** Alicia I. Hidron, Robert H. Gilman, Juan Justiniano, Anna J. Blackstock, Carlos LaFuente, Walter Selum, Martiza Calderon, Manuela Verastegui, Lisbeth Ferrufino, Eduardo Valencia, Jeffrey A. Tornheim, Seth O'Neal, Robert Comer, Gerson Galdos-Cardenas, Caryn Bern, for the Chagas Disease Working Group in Peru and Bolivia

**Affiliations:** 1 Division of Infectious Diseases, Department of Medicine, Emory University School of Medicine, Atlanta, Georgia, United States of America; 2 Asociacion Benefica PRISMA, Lima, Peru; 3 Facultad de Ciencias y Filosofía, Universidad Peruana Cayetano Heredia, Lima, Peru; 4 Hospital Universitario Japones, Santa Cruz de la Sierra, Bolivia; 5 Division of Parasitic Diseases, Centers for Disease Control and Prevention, Atlanta, Georgia, United States of America; 6 Atlanta Research and Education Foundation, Decatur, Georgia, United States of America; 7 Mount Sinai School of Medicine, New York, New York, United States of America; 8 Oregon Health Sciences University, Portland, Oregon, United States of America; 9 Wake Forest University Health Sciences, Winston-Salem, North Carolina, United States of America; National Institutes of Health, United States of America

## Abstract

**Background:**

Patients with Chagas disease have migrated to cities, where obesity, hypertension and other cardiac risk factors are common.

**Methodology/Principal Findings:**

The study included adult patients evaluated by the cardiology service in a public hospital in Santa Cruz, Bolivia. Data included risk factors for *T. cruzi* infection, medical history, physical examination, electrocardiogram, echocardiogram, and contact 9 months after initial data collection to ascertain mortality. Serology and PCR for *Trypanosoma cruzi* were performed. Of 394 participants, 251 (64%) had confirmed *T. cruzi* infection by serology. Among seropositive participants, 109 (43%) had positive results by conventional PCR; of these, 89 (82%) also had positive results by real time PCR. There was a high prevalence of hypertension (64%) and overweight (body mass index [BMI] >25; 67%), with no difference by *T. cruzi* infection status. Nearly 60% of symptomatic congestive heart failure was attributed to Chagas cardiomyopathy; mortality was also higher for seropositive than seronegative patients (p = 0.05). In multivariable models, longer residence in an endemic province, residence in a rural area and poor housing conditions were associated with *T. cruzi* infection. Male sex, increasing age and poor housing were independent predictors of Chagas cardiomyopathy severity. Males and participants with BMI ≤25 had significantly higher likelihood of positive PCR results compared to females or overweight participants.

**Conclusions:**

Chagas cardiomyopathy remains an important cause of congestive heart failure in this hospital population, and should be evaluated in the context of the epidemiological transition that has increased risk of obesity, hypertension and chronic cardiovascular disease.

## Author Summary

Latin America is undergoing a transition from disease patterns characteristic of developing countries with high rates of infectious disease and premature deaths to a pattern more like industrialized countries, in which chronic conditions such as obesity, hypertension and diabetes are more common. Many rural residents with Chagas disease have now migrated to cities, taken on new habits and may suffer from both types of disease. We studied heart disease among 394 adults seen by cardiologists in a public hospital in the city of Santa Cruz, Bolivia; 64% were infected with *T. cruzi*, the parasite that causes Chagas disease. Both *T. cruzi* infected and uninfected patients had a high rate of hypertension (64%) and overweight (67%), with no difference by infection status. Nearly 60% of symptomatic congestive heart failure was due to Chagas disease; mortality was also higher for infected than uninfected patients. Males and older patients had more severe Chagas heart disease. Chagas heart disease remains an important cause of congestive heart failure in this hospital population, but often occurs in patients who also have obesity, hypertension and/or other cardiac risk factors.

## Introduction

Chagas disease, caused by the parasite *Trypanosoma cruzi*, affects an estimated 8 million people in the Americas [1]. Transmission occurs via inoculation of *T. cruzi*-infected feces of the triatomine vector through the bite wound or mucosal surfaces. Infection may also be acquired congenitally, through blood transfusion, organ transplant or through consumption of contaminated food or drink. The acute phase is associated with high level parasitemia, and mild, non-specific symptoms in the majority of individuals [2]. After 8–12 weeks, infected individuals pass into the chronic phase, in which parasites are no longer detectable by peripheral blood microscopy and diagnosis relies on demonstration of anti-*T. cruzi* antibodies. Infection is life-long in the absence of successful treatment. Over a period of decades, 20–30% of infected individuals develop specific patterns of end-organ damage. The most common form, chronic Chagas cardiomyopathy, is characterized by conduction system abnormalities, brady- and tachyarrhythmias, dilated cardiomyopathy, apical aneurysm, and thrombus formation in the aneurysm or enlarged left ventricle [3]. Patients with Chagas heart disease have a high rate of mortality from ventricular arrhythmias, pulmonary or cerebral emboli, and intractable congestive heart failure [3].

Historically, *T. cruzi* transmission occurred predominantly in rural areas of Latin America where poor housing conditions promoted vector infestation. Since 1991, Chagas disease control programs have made striking progress in decreasing vector- and blood-borne *T. cruzi* transmission, leading to dramatic declines in infection prevalence among children [4], [5]. However, millions of *T.cruzi*-infected adults remain, and massive population movements over the past 3 decades have brought many of these individuals to cities across Latin America. Urban populations are in transition from an epidemiology of predominantly infectious diseases to patterns similar to industrialized countries where obesity, hypertension, diabetes and atherosclerosis are the leading causes of illness and death [6], [7]. Adults infected with *T. cruzi* as children form a transitional generation, experiencing the simultaneous impact of past infectious exposures and current cardiovascular risk factors.

Bolivia has the highest prevalence of *T. cruzi* infection in the world, estimated at 6% of the national population, and reaching 30–40% in surveys of pregnant women, blood donors or endemic community members [1], [8], [9], [10]. The major objective of this study was to assess *T. cruzi* cardiac morbidity and its coincidence with common cardiovascular risk factors and disease among patients attending a large urban public hospital. In addition, we explored risk factors for *T. cruzi* infection and disease severity, and clinical and epidemiological associations with positive results by *T. cruzi* PCR.

## Methods

### Ethics statement

The protocol was approved by the institutional review boards of the study hospital, Asociación Benéfica PRISMA, and the Centers for Disease Control and Prevention.

### Study site and patient population

The study was conducted in the Hospital Universitario Japonés in Santa Cruz, Bolivia from August 25 to November 13, 2008. The hospital is one of two public hospitals and serves approximately 60% of the city's uninsured population. Although the city of Santa Cruz does not have vector-borne T. cruzi transmission, infection prevalence is high because many residents migrated from rural areas with intense transmission. All patients attending the cardiology clinic, admitted to the cardiology inpatient service or seen by the cardiology service in inpatient consultations were eligible, with the exception of those younger than 18 years, pregnant, unable to provide informed consent or unable to undergo cardiac evaluation.

After written informed consent, a structured questionnaire was administered by a study nurse. Data included demographics and factors potentially associated with *T. cruzi* infection risk (e.g., type of housing, location of and infestation in residences throughout life, family history of sudden death and Chagas disease, history of transfusion). Each patient underwent a focused history and physical examination, electrocardiogram (EKG) and echocardiogram. The study physician and two cardiologists who performed the echocardiograms and interpreted the EKGs were blinded to patient infection status. Data on existing structural heart disease or risk factors for structural heart disease were collected from medical records, EKGs and echocardiograms. A 12-ml blood specimen was obtained from each participant, centrifuged and separated into serum and clot. In August 2009 (9 months after the completion of the cross-sectional study), telephone contact was attempted for all participants or their family members to ascertain whether the patients were alive. The date of death was sought for those no longer alive at that time.

### Laboratory methods

Serum specimens were screened for antibodies to T. cruzi using two commercial enzyme-linked immunosorbent assays (ELISA), one based on whole epimastigote lysate (Chagatek, bioMérieux, Buenos Aires, Argentina) and the other on recombinant antigens (Chagatest, Wiener laboratory, Rosario, Argentina). Results were interpreted following the manufacturers' instructions. For discordant results, an immunofluorescent antibody test (IFA) was performed using a titer of 1∶32 as the positive cut-off. A patient was considered to have confirmed *T. cruzi* infection if he or she had positive results by at least two serologic tests [11].

Polymerase chain reaction (PCR) was performed using 500 µl specimens of clot, based on an earlier analysis showing higher sensitivity in this specimen compared to buffy coat or whole blood [12].DNA was extracted following a standard phenol-chloroform extraction protocol [13].PCR amplifications were performed using the 121/122 primer set (5-AAATAA- TGTACGGGKGAGATGCATGA-3 and 5-GTTCGATTGGGGTTGGTGTAATATA-3) targeting the kinetoplast minicircle, using published methods and conditions [12], [14].A positive result was based on the appearance of the characteristic 330-bp product [12].

Quantitative real time PCR followed published methods [15].The primer set Cruzi 1 (5′–ASTCGGCTGATCGTTTTCGA–3′) and Cruzi 2 (5′–AATTCCTCCAAGCAGCGGATA–3′) was used to amplify a 166 base-pair DNA fragment. The probe Cruzi 3 (5′–CACACACTGGACACCAA–3′) was labeled with 5′FAM (6 – carboxyfluorescein) and 3′MGB (minor groove binder). TaqMan Human RNase-P detection reagent (Applied Biosystems) was included as an internal control. A result was considered valid only when the internal control was efficiently amplified. A non-template negative control was included in each run. A positive result was defined by the threshold cycle (Ct), the first cycle where fluorescence was detected above baseline. The C(t) was determined by the respective standard curve for the specimen batch, and was always between 37 and 38 cycles. A clot specimen was inoculated with 1×10^6^ *T. cruzi* Y strain trypomastigotes, extracted and diluted successively to determine the minimum quantity detectable; the limit was found to be 1 parasite/ml [8], [15].

### Clinical classifications

The American College of Cardiology and American Heart Association (ACC/AHA) heart failure classification system was applied for all patients with available echocardiograms [16]. For *T. cruzi*-infected patients, we also assigned a severity classification based on a slight modification of published methods:[17]

1. Stage A: *T. cruzi* infection with normal EKG and echocardiogram
2. Stage B: *T. cruzi* infection with EKG abnormalities and/or minor echocardiogram abnormalities (segmental wall motion abnormalities, left atrial dilatation, apical aneurysm, diastolic dysfunction) with preserved systolic function (ejection fraction (EF) ≥55%) and no left ventricular dilatation
3. Stage C: *T. cruzi* infection with mild to moderate systolic dysfunction (EF 40–54%) and/or left ventricular dilatation
4. Stage D: *T. cruzi* infection with severe systolic dysfunction (left ventricular end diastolic diameter ≥57 mm, EF ≤40% and NYHA classification III or IV).

Stages were not assigned for infected subjects whose clinical and echocardiogram findings indicated an etiology other than Chagas cardiomyopathy. All other *T. cruzi*-infected patients with echocardiogram data were assigned a Chagas cardiomyopathy stage, regardless of the presence or absence of comorbidities.

Hypertension, diabetes, coronary disease were defined based on patient history and/or physician documentation in the medical record chart. Body mass index (BMI) was calculated (weight in kilograms divided by the square of the height in meters).

### Statistical analysis

Cross-sectional analyses were performed in SAS version 9.2 (SAS Institute) for three outcome measures: (1) *T. cruzi* infection among all subjects; and (2) positive *T. cruzi* PCR results and (3) Chagas cardiomyopathy severity stage among *T. cruzi*-infected subjects only. Categorical variables were compared by the exact Pearson's chi-square test; continuous variables were analyzed using the Wilcoxon rank-sum test. Variables significant at α = 0.05 were considered for multivariable logistic regression models, and appropriate 2-way interactions were tested.

## Results

### Study population and clinical status

Of 549 patients seen by the cardiology service during the study period, 394 (72%) enrolled in the study. Three excluded patients were pregnant, 8 were younger than 18 years, 44 declined participation, and 100 left the hospital prior to contact with the research staff. One patient failed to meet the serological criteria for confirmed infection (positive results by Wiener ELISA, negative by Chagatek ELISA and IFA) but had positive results by *T. cruzi* PCR; this patient was excluded from the epidemiological analyses. Of the 393 patients included in the analysis, 65% were female. The mean age was 52.0 years (range 20–86) for females and 51.4 years (18–87) for males. The prevalence of confirmed *T. cruzi* infection was 63.9% (251/393). Overall, 64% of participants had a history of hypertension and 67% were overweight or obese (defined as body mass index [BMI] >25). Other markers or risk factors for structural heart disease were less common. Of the 342 patients with ACC/AHA staging data, 155 (45%) had stage C or D, indicating symptomatic congestive heart failure; of these, 91 (58.7%) had Chagas cardiomyopathy, whereas 23 (14.8%) had *T. cruzi* infection but another probable etiology for their cardiac insufficiency.

Chagas cardiomyopathy severity stage was assigned for 191 *T. cruzi*-infected patients: 27 (14.1%) stage A (equivalent to indeterminate), 97 (50.8%) stage B, 51 (26.7%) stage C and 16 (8.4%) stage D. Chagas severity stage was not determined for the 38 *T. cruzi*-infected patients whose heart disease was judged to be from another etiology. This group included 2 patients with isolated left ventricular hypertrophy (LVH), 2 with left atrial dilatation associated with severe mitral stenosis or mitral valve prolapse, 26 with diastolic dysfunction and/or LVH due to hypertensive disease, 4 with segmental hypokinesis and systolic dysfunction in setting of an acute coronary syndrome, and 1 each with a pericardial tumor and congenital heart disease. Twenty-two infected patients were lacking echocardiogram data and could not be assigned a Chagas severity stage for that reason.

Of the 393 patients in the analysis, survival status 9 months after completion of the cross-sectional study was determined for 325 (83%); 25 (7.7%) patients died during that time, but dates of death were only available for 19, precluding a formal survival analysis. Patients with *T. cruzi* infection were more likely to have died than those without infection (21/210 (10%) versus 4/115 (3%); *p* = 0.05). However, the strongest predictor of death was ACC/AHA heart failure stage: 19 (15%) of 125 patients with stage C or D died, compared to 0 (0%) of 59 with stage A and 1 (1%) of 102 with stage B (*p*<0.001). Of the 19 patients with ACC/AHA stage C or D, 15 (79%) had Chagas disease; the one deceased patient with ACC/AHA stage B also had Chagas disease. Five of the deceased patients, all with *T. cruzi* infection, had not had echocardiograms and therefore were missing ACC/AHA stage data.

### Associations with *T. cruzi* infection

Males and females were equally likely to have *T. cruzi* infection (Table 1). Infected patients were older than uninfected patients (mean 53.7 vs. 48.3 years, *p* = 0.001). *T. cruzi* infection prevalence increased with age to a peak at approximately 58 for males and 69 for females, and then declined, demonstrating a significant quadratic relationship. Age was therefore included as a quadratic function in models with *T. cruzi* infection as the outcome.

**Table 1:** Characteristics of seropositive and seronegative study participants.

Characteristic	Seropositive (N = 251) n (%)	Seronegative (N = 142) n (%)	P value (Unadjusted)^a^	P value (Adjusted)^b^
**Gender**				
Male	90 (35.9)	48 (33.8)	0.7417	0.4092
Female	161 (64.1)	94 (66.2)	…	…
**Type of recruitment**				
Inpatient cardiology service/consultation	62 (24.7)	20 (14.1)	0.0140	0.0107
Outpatient clinic	189 (75.3)	122 (85.9)	…	…
**Structural heart disease and/or risk factors**				
Hypertension c	149 (64.0)	86 (64.2)	1.0000	0.1148
Coronary artery disease	37 (14.7)	25 (17.6)	0.4734	0.0731
Diabetes^d^	42 (19.7)	20 (16.5)	0.5586	0.6277
Arrhythmia	49 (19.5)	13 (9.2)	0.0091	0.0286
Body mass index ≥25^e^	154 (69.4)	83 (61.9)	0.1648	0.7889
**ACC/AHA Heart Failure Classification** f				
Stage A	49 (21.4)	16 (14.2)	0.0007	<0.0001
Stage B	66 (28.8)	56 (49.6)	…	…
Stage C or D	114 (49.8)	41 (36.3)	…	…

^**a**^Unadjusted results based on exact Pearson chi-square tests.

^**b**^Adjusted results based on logistic regression, adjusted for gender and age as a quadratic function. Note that gender is adjusted for age as a quadratic function only. See text for explanation of age adjustment.

^**c**^Data missing for 18 seropositive and 8 seronegative patients.

^**d**^Data missing for 38 seropositive and 21 seronegative patients.

^**e**^Data missing for 29 seropositive and 8 seronegative patients.

^**f**^Data missing for 22 seropositive and 29 seronegative patients.

The prevalence of hypertension, coronary artery disease, diabetes and BMI >25 did not differ among *T. cruzi*-infected versus uninfected patients (Table 1). There were no significant differences in findings for overweight (BMI 25–29.9) compared to obese patients (BMI >30). We therefore used a combined category that included overweight and obesity. In both unadjusted and adjusted analyses, *T. cruzi*-infected patients had significantly higher prevalence of arrhythmias and severe congestive heart failure as measured by ACC/AHA classification, and were more likely to have been recruited from the inpatient service compared to uninfected patients. In the model adjusted for age and sex, infected patients were less likely to report coronary artery disease; this difference was borderline significant. There were no differences by infection status in the frequency of valvular heart disease, congenital heart disease, stroke, or transitory ischemic attacks, or of reported symptoms such as dizziness or lightheadedness, syncope, palpitations, typical or atypical chest pain, exertional dyspnea, dysphagia, or constipation (data not shown, but available upon request). Infected and uninfected patients were equally likely to have an implanted pacemaker (14/251 versus 5/142; *p* = 0.47). In models adjusted for age and sex, *T. cruzi-*infected patients were significantly more likely than uninfected patients to have a right bundle branch block (RBBB), left ventricular dilatation, ‘pure’ left atrial dilatation (defined as left atrial end diastolic diameter >40 mm not explained by diastolic dysfunction or LVH), low ejection fraction, diffuse wall motion abnormalities, apical aneurysm or intracavitary thrombi, and less likely to have LVH (Table 2).

**Table 2:** EKG and echocardiogram findings among patients with and without *Trypanosoma cruzi* infection.

	Seropositive n (%)	Seronegative n (%)	P value (Unadjusted)^a^	P value (Adjusted)^b^
**EKG findings** c				
Atrial fibrillation/flutter	25 (11)	6 (4)	0.0501	0.0938
Any ventricular arrhythmia^d^	13 (5)	3 (2)	0.1864	0.3758
Left anterior fascicular block	28 (12)	11 (8)	0.2941	0.4448
Right bundle branch block	41 (17)	9 (7)	0.0041	0.0116
Bifascicular (right bundle and left anterior fascicular) block blocks)^e^	19 (8)	3 (2)	0.0221	0.1107
Abnormality 1^st^ portion of QRS	55 (23)	17 (12)	0.0139	0.0568
Left ventricular hypertrophy	21 (9)	22 (16)	0.0433	0.0500
**Echocardiogram findings** e				
Left ventricular dilatation^f^	50 (22)	13 (10)	0.0087	0.0114
“Pure” left atrial dilatation^g^	43 (19)	14 (11)^h^	0.0696	0.0296
Ejection fraction <55%	62 (27)	18 (14)	0.0053	0.0162
Diffuse wall motion abnormality	39 (17)	8 (6)	0.0050	0.0087
Apical aneurism	23 (10)	1 (1)	0.0006	0.0187
Intracavitary thrombus	22 (10)	2 (2)	0.0033	0.0279
Left ventricular hypertrophy	49(21)^h^	38 (30)	0.0940	0.0045
Diastolic dysfunction	112 (49)	61 (48)	0.9121	0.1319

a Unadjusted results based on exact Pearson chi-square tests.

b Adjusted results based on logistic regression, adjusted for sex and age as a quadratic function. See text for explanation of age adjustment.

c EKG analyses include a total of 237 seropositive and 137 seronegative participants. 14 seropositive and 5 seronegative participants were excluded from the EKG analyses because they had implanted pacemakers.

d Denominator for ‘any ventricular arrhythmia’ includes 14 seropositive and 5 seronegative participants with pacemakers excluded from the other EKG analyses.

e Echochardiogram analyses include 229 seropositive and 127 seronegative participants.

f Defined as end diastolic diameter >57 mm.

g Defined as left atrial end diastolic diameter >40 mm not explained by diastolic dysfunction or left ventricular hypertrophy.

h Data missing for one participant.

In a multivariable logistic regression model, rural residence, poor housing conditions and increasing duration of residence in an endemic province were associated with increased risk of *T. cruzi* infection, with a significant interaction between the latter two variables (Table 3). For each additional decade living in an endemic province, people who lived in poor housing experienced a 3.2-fold increase risk of *T. cruzi* infection compared a 1.7-fold increase for those who lived in moderate or good housing.

**Table 3:** Multivariable logistic regression model of factors associated with risk of *Trypanosoma cruzi* infection.

Risk Factors^a^	Odds Ratio	95% Confidence Intervals
Gender (male vs. female)	1.25	0.73–2.15
Ever lived in rural area	2.07	1.12–3.83
Ever lived in poor housing (house with both adobe walls and dirt floor) a	2.65	1.51–4.65
10-year increase in residence in an endemic province^b^		
Among those with good to intermediate housing	1.71	1.37–2.14
Among those with poor housing	3.20	2.14–4.78

Data missing for 7 patients.

^**a**^Results for each risk factor are adjusted for other variables listed and for age as a quadratic function; see text for explanation.

^**b**^Significant interaction between years of residence in an endemic area and housing. Risk associated with housing conditions evaluated holding years in endemic area fixed at the mean (45.2 years).

### Severity of Chagas cardiomyopathy

In univariate analyses, increasing age (odds ratio [OR] per 10-year increase in age 1.40; 95% confidence interval [CI] 1.09–1.81), male sex (OR 5.87; 95% CI 3.04–11.31), having lived in poor housing (OR 2.46; 95% CI 1.29–4.71) and decades living in an endemic province (OR 1.24; 95% CI 0.99–1.54) were associated with elevated risk of severe disease, defined as Chagas cardiomyopathy stage C or D. Having BMI >25 was associated with a trend to decreased risk of severe disease (OR 0.56; 95% CI 0.29–1.10). In a multivariable model, male sex, increasing age and poor housing remained strong predictors of severe disease (Table 4). In the adjusted models, no significant association was detected for other variables (including BMI >25, hypertension, coronary artery disease, or diabetes, positive results by PCR, prior treatment for *T. cruzi* infection, and cumulative time living in an infested house or rural area).

**Table 4:** Multivariable logistic regression model of factors associated with severity of Chagas cardiomyopathy, defined as Chagas stage C or D (versus Chagas stage A or B).

Factor	Odds Ratio	95% Confidence Intervals
Male sex	7.06	3.44–14.47
10-year increase in age	1.54	1.15–2.07
Ever lived in a house with both adobe walls and dirt floor	2.23	1.08–4.62

### Associations with positive results by *T. cruzi* PCR

Of 251 patients with confirmed positive results by serology, 109 (43%) had positive results by conventional PCR, and of these, 89 (81.7%) also had positive results by real time PCR. Among specimens with positive results by real time PCR, the median number of parasite copies/mL was 79.4 (range 1.1 – 313,330). One patient (excluded from the epidemiological analyses) had positive results by 1 of 3 serological tests, but positive results by conventional and real time PCR. If this patient is considered uninfected based on the serological results, both conventional and real time PCR had calculated specificities of 99.3% (142/143). Specimens with positive results by PCR had higher mean Chagatek and Wiener ELISA absorbance values (defined as optical density minus ELISA plate cut-off value) than those with negative PCR results (median Chagatek absorbance 1.34 vs. 1.23, p = 0.006; Wiener 2.28 vs. 2.21, p<0.001). However, the distribution of absorbance values for specimens positive and negative by PCR had a high degree of overlap. There was no difference in ELISA absorbance values by Chagas severity stage (*P* = 0.73).

Epidemiological associations with positive results by conventional PCR and real time PCR were similar. Among seropositive participants, males were significantly more likely than females to have positive results by conventional PCR (OR 2.16; 95% CI 1.28–3.65) or by real time PCR (OR 1.830; 95% CI 1.03–3.26). In additional univariate analyses, positive conventional PCR results were less frequent among patients with BMI >25 (OR 0.40; 95% CI 0.22–0.71), a history of hypertension (OR 0.54; 95% CI 0.31–0.92) or signs of left atrial dilatation on echocardiogram (OR 0.50; 95% CI 0.29–0.85). There was no association between severity of heart failure or Chagas cardiomyopathy and positive results by conventional or real time PCR. In a multivariable logistic regression model adjusted for age and disease severity, male sex remained associated with higher odds and BMI >25 with lower odds of positive results by conventional PCR (Table 5). Parasite load as measured by real time PCR was higher among males than females (mean copies/mL 4292 vs 1286, *P* = 0.0032), but there was no difference by BMI category (*P* = 0.94).

**Table 5:** Multivariable model of characteristics associated with positive results on conventional *Trypanosoma cruzi* PCR among seropositive patients.

Factor	Odds Ratio	95% Confidence Intervals
10-year increase in age	0.94	0.72–1.22
Male sex	2.51	1.17–5.36
Chagas stage C/D (vs. A/B)	0.67	0.31–1.47
Overweight or obese (BMI ≥25)	0.44	0.22–0.87

Data missing for 96 patients.

## Discussion

A profound epidemiological and nutritional transition has been underway in Latin America for the last several decades [7], [18], [19]. In some countries, such as Chile and Uruguay, a pattern of non-communicable disease epidemiology similar to the United States or Europe is now predominant [18]. In other countries, such as Guatemala and Bolivia, the rural population still lives in pre-transitional conditions with limited access to adequate housing, diet, clean water and sanitation [20]. However, when the rural poor migrate to cities, they often experience an abrupt transition to more sedentary lives and calorie-dense diets, and may add obesity, diabetes and hypertension to pre-transition conditions, including Chagas disease [21].

Our data offer a graphic illustration of the challenge facing health care systems in *T. cruzi*-endemic areas. We found that 59% of congestive heart failure cases were attributable to Chagas cardiomyopathy, and 79% of deaths in patients with advanced congestive heart failure occurred in those with Chagas disease. These figures are much higher than the 6–22% of congestive heart failure cases and 8% of congestive heart failure deaths attributed to Chagas disease in facility-based data from Brazil and Argentina reviewed in a recent publication [22], and illustrate the very high disease burden still caused by *T. cruzi* in Bolivia today. Nevertheless, a majority of both *T. cruzi*-infected and uninfected patients in Santa Cruz had additional cardiovascular risk factors such as hypertension, obesity and diabetes. Although the increasing burden of chronic non-infectious disease in developing countries is well recognized [22], there are no published data regarding potential interactions of these conditions with infectious diseases such as *T. cruzi*. Our data represent a first snapshot of their co-occurrence and highlight issues that merit further investigation, including more comprehensive assessment of cardiovascular risk factors and co-morbidities, population-based disease burden assessments, and longitudinal follow-up to evaluate prognosis and disease progression over time.

Our study suffers from the inherent limitations of facility-based data: patterns may reflect differences in health care seeking behavior rather than biological differences, and the magnitude of the morbidity burden does not reflect what would be found in a population-based study because patients with more severe disease are more likely to seek care than those with milder disease. Consistent with the published literature [23], [24], [25], we found male sex to be associated with a substantially increased risk of severe Chagas heart disease. As expected from the chronic progressive nature of the cardiomyopathy, severity also increased steadily with age. The fall-off in seroprevalence among older adults in endemic communities has long been attributed to excess mortality among infected individuals as their heart disease worsened after age 45 [26], [27]. Survival is markedly shortened among Chagas cardiomyopathy patients once they have heart failure or other indicators of advanced cardiomyopathy [25], [28], [29]. We also observed similar age- and sex-related patterns. However, because our data are facility-based, we cannot rule out the possibility that they reflect differences in health care access.

Morbidity and mortality from Chagas disease tend to occur at an older age now than previously [30], and this may be reflected in the older age of peak seroprevalence in our data compared to earlier studies. In part, this represents a cohort effect: as vector control has decreased exposure and infection incidence among younger people, Chagas disease is becoming a disease of older adults [30]. In addition, better clinical management and health care access may have improved survival, especially for those living in urban areas [31]. Many experts also believe that intense exposure to infected vectors and repeated *T. cruzi* reinfection contributed to accelerated progression of cardiomyopathy and high rates of severe disease in the decades before the Southern Cone Initiative began in 1991, and that vector control has led to an amelioration of Chagas cardiomyopathy since then [32], [33], [34]. Although this hypothesis is difficult to test in human populations, animal models provide supporting evidence that repeated infection worsens the long-term prognosis of Chagas heart disease [35]. We observed that patients who had lived in a house with earthen floor and walls were more likely to have severe Chagas cardiomyopathy; this finding may reflect poor housing conditions acting as a proxy for more intense vector exposure and reinfection risk.

Our clinical findings are consistent with many published studies, demonstrating associations with right bundle branch block, left ventricular dilatation, low ejection fraction, apical aneurysms, and intracavitary thrombi [3]. The risk factors for infection identified in our data were not surprising, and as shown in many previous studies, included markers for or consequences of poverty, such as lower educational level, rural upbringing and having lived in houses with adobe walls and earthen floors. The intensification of risk seen when poor quality housing was combined with longer residence in an endemic province underscores the importance of maintaining both vector control and housing improvement programs in rural Bolivia, where infestation has not yet been eliminated [36].

We found no consistent association between parasite burden as indicated by positive PCR results and disease severity; this negative finding may result from the limitations of the cross-sectional design or the molecular methods, or may reflect the pathophysiology of the disease. In the last 10 years, a consensus has emerged that parasite persistence underlies the development and progression of Chagas cardiomyopathy [34], [37]. In a study of left ventricular heart muscle tissue from necropsies of patients who died with different stages of chronic Chagas cardiomyopathy, a positive association among inflammation, fibrosis and parasite DNA was found [38]. Other authors endorse the importance of parasite persistence in tissue the pathogenesis of chronic Chagas' cardiomyopathy [39], [40] Nevertheless, demonstration of a quantitative link between parasite burden and cardiac pathology or disease severity has been elusive [34]. This failure may be due in part to the limitations of existing detection methods. PCR assays, while more sensitive that culture or xenodiagnosis, are imperfect [41]. Sampling biases may also exist: when multiple specimens are examined, whether by xenodiagnosis, culture or PCR, many individuals have detectable parasitemia on some occasions but not others and parasite levels in peripheral blood may not correlate well with parasite loads in target tissues such as the heart [41], [42]. In a life-long infection such as Chagas disease, the question of timing is also essential. In theory, the impact of parasite load on pathology could occur early but with enduring effects. In an earlier study of 56 infected individuals, positive PCR results at baseline was predictive of increased progression risk over a several-year period of longitudinal follow-up [23]. In contrast, other studies have failed to show any association between the presence or level of parasitemia and cardiomyopathy progression [24], [41], [42].

We found intriguing epidemiological associations with *T. cruzi* PCR results. Males were significantly more likely to have positive PCR results than females, and obesity was associated with a lower prevalence of positive results. Animal models suggest that male gonadal hormones decrease immune control of the parasite [43]. Orchiectomy of male mice enhances clearance of *T. cruzi* infection and reduces the amastigote burden in heart tissue, whereas parasitemia levels increase when castrated mice receive supplemental testosterone. In female mice, the parasitemia level rises after oophorectomy, and can be suppressed by estrogen and/or progesterone supplementation [44]. To our knowledge, the association of obesity with lower likelihood of positive results by *T. cruzi* PCR has not previously been reported in a clinical study. However, mouse models of chronic *T. cruzi* infection reveal large numbers of parasites in adipocytes. Indeed, more parasites are seen per adipocyte than in the tissues such as heart where parasites are classically thought to reside during chronic infection, suggesting that adipose tissue may represent a long-term reservoir and potentially a parasite sink [45]. Further investigation is needed to confirm the validity of these epidemiological observations and their physiological significance.

One of our study participants had discordant serology and positive PCR results; we excluded her from the epidemiological analyses, because her results did not meet the standard criteria for confirmed chronic *T. cruzi* infection, which require at least 2 positive serological tests [11]. Nevertheless, we believe these results reflected a true infection. Positive PCR results in specimens from seronegative individuals have been reported in a number of studies, reflecting the fact that none of the diagnostic options for chronic *T. cruzi* infection has perfect sensitivity and specificity, and any given set of test results should be interpreted with care [41], [46], [47], [48].

As control programs decrease *T. cruzi* incidence, attention is shifting to the large number of adults infected before these programs began. At the same time, *T. cruzi*-endemic countries are experiencing population shifts, urbanization and the epidemiological transition. Better understanding of the role of cardiac comorbidities and metabolic factors in the course and evolution of chronic Chagas cardiomyopathy will be essential to optimizing management of these patients in the future.
